# Full Mouth Rehabilitation with All-Ceramic Restorations in a Patient with Amelogenesis Imperfecta: A Case Report with 10-Year Follow-Up

**DOI:** 10.3390/dj13120546

**Published:** 2025-11-21

**Authors:** Stefanos Kourtis

**Affiliations:** Department of Prosthodontics, Dental School, National and Kapodistrian University of Athens, 11527 Athens, Greece; stefkour@dent.uoa.gr

**Keywords:** amelogenesis imperfecta, all-ceramic restorations, full mouth rehabilitation

## Abstract

**Background:** Amelogenesis imperfecta (AI) includes a group of inherited disorders that affect enamel formation, both in quality and quantity. It may cause anomalies in a number of teeth or a group of teeth, or it may be present in the whole dentition. The main complaints of patients who are affected by AI are increased sensibility to hot and cold food, impaired esthetic appearance, discoloration of mandibular and maxillary anterior teeth, and masticatory problems. The treatment of adult patients with amelogenesis imperfecta usually demands a multidisciplinary approach because several problems are present. Pediatric treatment is usually undertaken at an early stage, and orthodontic treatment usually begins in adolescence. Periodontal and prosthetic treatments are usually required for the rehabilitation of patients who usually have been expecting this treatment for years. **Objective:** The aim of this case report is to present a full mouth rehabilitation with all-ceramic restorations in a young patient with amelogenesis imperfecta, with follow-up at 10 years. **Treatment:** An 18-year-old with amelogenesis imperfecta presented for functional and esthetic rehabilitation. The patient underwent a second orthodontic treatment, conservative periodontal therapy, and restored with all-ceramic restorations. **Results:** The patient was fully satisfied with the outcome of the therapy, and the clinical situation remained stable at 10-year recall. **Conclusions:** All-ceramic restorations can be a clinically acceptable option for the rehabilitation of patients with amelogenesis imperfecta.

## 1. Introduction

Amelogenesis imperfecta (AI) includes a group of inherited disorders that affect enamel formation, both in quality and quantity. It may cause anomalies in a number of teeth or a group of teeth, or it may be present in the whole dentition. Often, amelogenesis imperfecta also co-exists with other dysplasia of dental tissues, for example, dentinogenesis imperfecta [1,2].

The prevalence of AI varies. In the United States, prevalence is estimated at 0.06 to 0.07:1000 individuals. In a northern Swedish county, AI prevalence was estimated to be as high as 1.4:1000 individuals. These differences are mainly due to diagnostic and demographic criteria, along with related mutant genes, in studied populations [3,4].

Amelogenesis imperfecta belongs to a genetically heterogeneous group of inherited enamel defects that affect the formation and mineralization of dental enamel. Its etiology is primarily rooted in mutations of genes involved in enamel matrix formation, processing, and mineralization. Rarely, enamel defects resembling AI may be associated with environmental factors, but true AI is considered a genetic disorder [1,5,6].

Intraoral clinical features of hypomaturated AI include abnormally rough and pitted tooth surfaces, discoloration of teeth, and hypersensitivity. These abnormal tooth surfaces attract plaque and calculus, causing teeth to be at a high risk of caries and attrition at a young age. Defective or absent enamel may cause tooth hypersensitivity. Therefore, full-coverage restorations are often needed for the function, comfort, and esthetic appearance of the patient [1,3].

Additional clinical characteristics of AI may also include short clinical crown heights, malformed teeth, congenitally missing teeth, supernumerary teeth, pulp calcifications, taurodontism, root malformations, anterior open bite, and abnormal growth patterns of the maxilla and the mandible [7,8].

Numerous classifications for amelogenesis imperfecta have been proposed. Based on phenotypes and inheritance, up to 15 subtypes have been introduced. Four main types of amelogenesis imperfecta have been distinguished based on clinical and radiographic characteristics [1,9,10], as follows:

- Hypoplastic, where enamel is well mineralized but reduced in quantity;
- Hypomaturated, where the mineralization is abnormal;
- Hypocalcified, where enamel is normally formed but undermineralized;
- Combination of the above-mentioned types.

The surfaces of affected teeth can be rougher than normal, and surface hardness is reduced. Typically, the still-existing enamel shows marked discoloration. In subjects with hypoplastic AI, affected teeth characteristically exhibit a rough pitted surface with grooves, along with a reduced thickness of the enamel layer due to deficiencies in the building of the enamel [8,10]. In the case of hypocalcified AI, the hardness of enamel is reduced but the enamel layer is of normal thickness, and the enamel is often yellow-brownish discolored. The enamel layer in patients with hypomaturated forms of AI is of normal thickness, but the enamel is softer and is often chipped away [11,12,13].

Patients with amelogenesis imperfecta on the permanent dentition usually have orthodontic problems and sometimes also anterior open bite [14,15]. The main complaints of patients who are affected by AI are increased sensibility to hot and cold food, impaired esthetic appearance, discoloration of mandibular and maxillary anterior teeth, and masticatory problems [11,12,13].

The impact of amelogenesis imperfecta on the quality of life of patients was investigated in a clinical study which found that in 90% of patients the main complaint was discoloration of their teeth, with 77% asking for an improvement in their smile. For nearly the same percentage (74%), reduction in sensitivity was the most important reason for seeking dental treatment, while improvement in tooth size was important for 60% of patients [16].

The treatment of young adult patients with amelogenesis imperfecta usually demands a multidisciplinary approach because several problems are present. Pediatric treatment is usually undertaken at an early stage, and orthodontic treatment usually begins in adolescence. Periodontal and prosthetic treatment is usually required for the rehabilitation of patients who usually have been expecting this treatment for years [17].

Despite the numerous publications on AI, there is no established standard clinical protocol for restoration in patients with amelogenesis imperfecta, and most published data refers to case reports or case series. In every clinical case, however, a detailed treatment plan should be made for each patient individually, taking into consideration the gravity of the dental lesions, the longevity of the planned restoration, and a prediction of the final result with maximum accuracy.

In this case report a prosthetic restoration with all-ceramic restorations in a young patient with amelogenesis imperfecta is presented, with follow-up at 10 years.

## 2. Case Presentation

***Background:*** An 18-year-old Caucasian patient presented for treatment of his dentition at the author’s private practice. The patient was in full health, and no systemic disease was reported. He was not taking any medicaments systematically, and he was not a smoker. The main complain of the patient was a psychological sense of “shame” because of the appearance of his teeth. Amelogenesis imperfecta had been diagnosed at a very early age, and the patient had received regular preventive and supportive dental treatment from a pediatric dentist. Prefabricated metal crowns had been placed over the first molars several years previously to prevent further damage of these permanent teeth. All teeth showed surface anomalies, discolorations, and irregular enamel surfaces with chippings and abrasions; in addition, all teeth had reduced dimensions.

**Figure 1:**
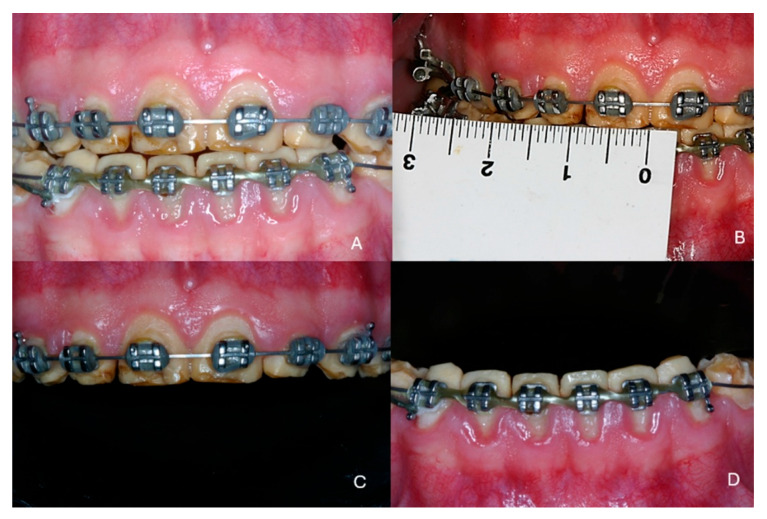
Initial clinical situation at the end of the first orthodontic treatment. (**A**) frontal view, (**B**) hypoplastic teeth with reduced dimensions, (**C**) maxillary arch, (**D**) mandibular arch.

The patient had been referred for orthodontic treatment two years previously without any prior prosthodontic consultation. The teeth were brought into proximity, and all diastemas were closed. As a result, for this patient, the available space for the teeth in the restoration would be limited, and the teeth would not be of the normal size (Figure 1A–D). Additionally, gingival inflammation was noticed which required immediate periodontal treatment.

***Diagnostic assessment/Initial intervention:*** After initial clinical and radiographic examinations, the latter carried out with a panoramic X-ray, the patient was referred for a second orthodontic treatment. The aim of the second treatment was to create adequate symmetrical space between the teeth to allow restoration with teeth in proper dimensions corresponding to the face of the patient. Extraction of impacted third molars was also performed to avoid any rebound of the orthodontic treatment.

**Figure 2:**
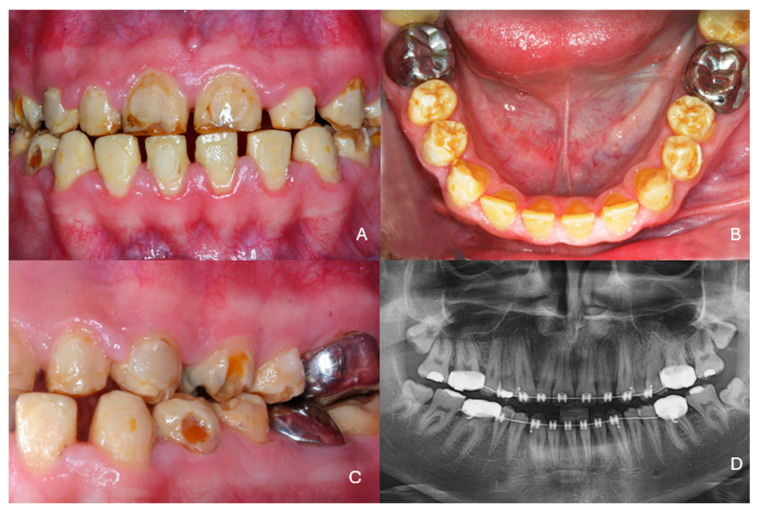
Clinical situation at the end of the second orthodontic treatment. (**A**) frontal view, (**B**) occlusal view of the mandibular teeth, (**C**) side view, (**D**) radiographic examination.

***Therapeutic intervention:*** At the end of the second orthodontic treatment diastemas between the teeth had been intentionally created so that the teeth in the restorations would have the desired size. Increasing the vertical dimension was also necessary, as several teeth were hypoplastic and abraded. Severe inflammation of the gingival tissues was noticed, and the patient was referred for periodontal treatment (Figure 2A,B).

Generalized gingival inflammation was noticed, and the interdental papillae were hyperplastic. Conservative periodontal treatment was performed as no pockets or marginal bone loss was found. At the periodontal recall the sulcus depth was max 3–4 mm and no mobility was noticed. All teeth were vital.

A thorough clinical examination of the teeth revealed characteristic discoloration, irregular enamel surfaces, and abfractions (Figure 2C). Radiographic examination confirmed the characteristics of amelogenesis in all teeth (Figure 2D).

The esthetic appearance of the dentition had severely influenced the patient psychologically since childhood. As he reported at the initial appointment, he always hesitated to smile, his smile was restricted, he always covered his mouth when laughing, and he had low self-confidence.

**Figure 3:**
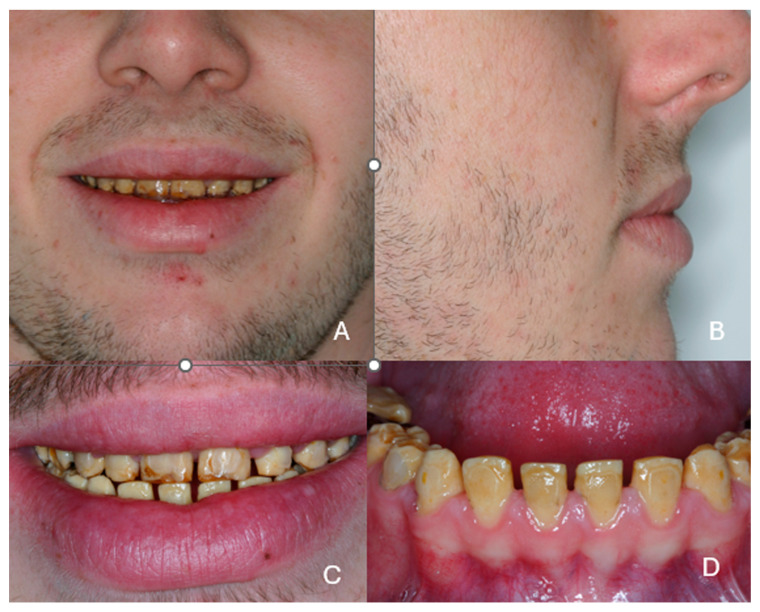
(**A**,**B**) Face and side photos of the patient at the end of the second orthodontic treatment, (**C**) Restricted smile of the patient, (**D**) The mandibular anterior teeth with diastemas.

The vertical dimension of occlusion was also reduced (Figure 3A,B). The patient showed a restricted smile and avoided exposing his teeth (Figure 3C,D). As a result, his social life was restricted to his close-family environment, and he presented symptoms of agoraphobia. His main concern, which he expressed intensely from the first visit, was, in his own words *“…to have natural and nice teeth as everybody”.*

**Figure 4:**
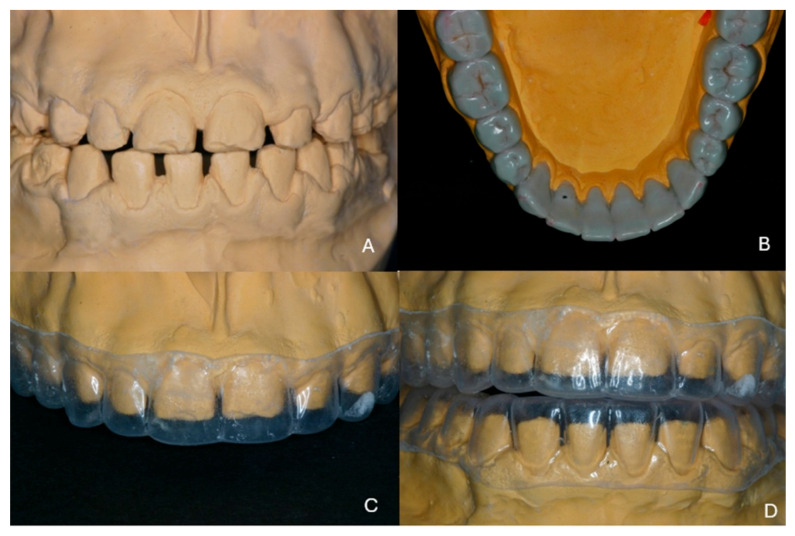
(**A**) Mounted study casts in the existing maximal intercuspation, (**B**) wax-up of the mandibular teeth, (**C**,**D**) thermoplastic sheets as copies of the wax-up.

At the end of the second orthodontic treatment the patient was re-evaluated prior to the restorative procedure. Diastemas were left between the teeth to ensure the proper dimensions of teeth in the restoration. Study casts were fabricated and mounted on a semi-adjustable articulator (Figure 4A). Face-bow registration was performed and the centric relation was recorded using an anterior deprogrammer. The vertical dimension of occlusion was increased by 3 mm to ensure adequate prosthetic vertical space. A detailed wax-up was created for all maxillary and mandibular teeth in the new vertical dimension (Figure 4B). The wax-up was presented to the patient, and he agreed immediately to the planned restoration.

On a duplicate model of the wax-up a thermoplastic sheet was formed for use as a guide during preparation of the teeth to ensure proper space for the restorations (Figure 4C,D). A mock-up would have been more helpful, but at the time of the treatment (10 years ago) this technique was not so widespread in clinical practice.

On the study casts the teeth were prepared initially for the laboratory fabrication of provisional restorations. In the presented case, the wax-up was scanned and provisional restorations were designed and fabricated as shells by milling using the CAD/CAM technology that was available at that time.

**Figure 5:**
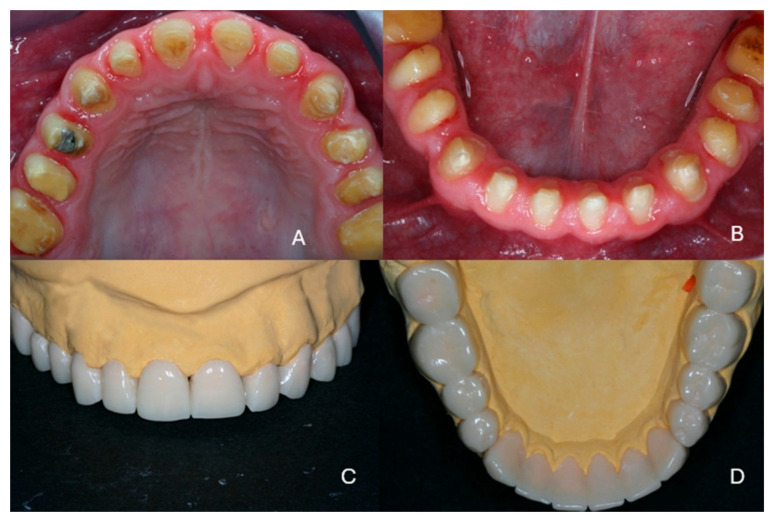
(**A**,**B**) Preparations of the maxillary and mandibular teeth, (**C**,**D**) milled provisional restorations.

The teeth were prepared with a circumferential shallow chamfer using the thermoplastic sheet to control the depth and avoid any unnecessary sacrifice of the dental tissues (Figure 5A–D). An endodontic treatment was needed on the second right mandibular molar because the patient complained about intense sensitivity after the tooth preparation.

**Figure 6:**
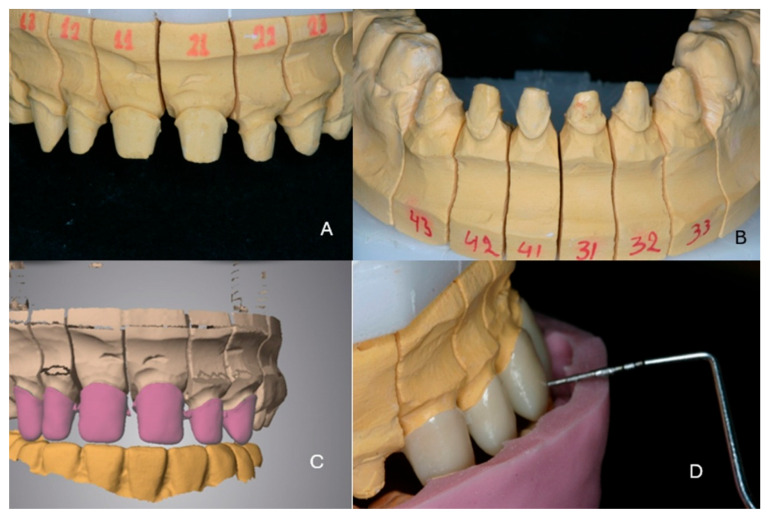
(**A**,**B**) The stone working casts, (**C**) digital design of the zirconia frameworks, (**D**) control of the available space for the veneering material.

Conventional impressions were obtained using A-Silicone (Elite, Zhermack Co., Rovigo, Italy) impression material, and working casts were fabricated from dental stone. A new face-bow registration was performed, and centric relation was recorded on the previously established increased vertical dimension which was applied for the provisional restorations using an anterior deprogrammer. Color shading was selected in accordance with the patient’s wishes. As a zirconia framework would be used, the discolored underlying dental tissue would not affect the final shade of the restorations. The stone casts were scanned in a laboratory scanner, and zirconia frameworks were designed using dedicated software (Exocad, Exocad Co., Darmstad, Germany). The frameworks were fabricated by milling of zirconia disk (Vita YZ, Vita Co., Bad Saeckingen, Germany) in a CAD/CAM device (imes icore 450i, imes-icore Co., Eiterfeld, Germany) and sintered according to the manufacturer’s instructions. The thickness of the ceramic framework was 0.8–1 mm, and the remaining space for veneering was checked with a silicone partial impression from the wax-up (Figure 6).

**Figure 7:**
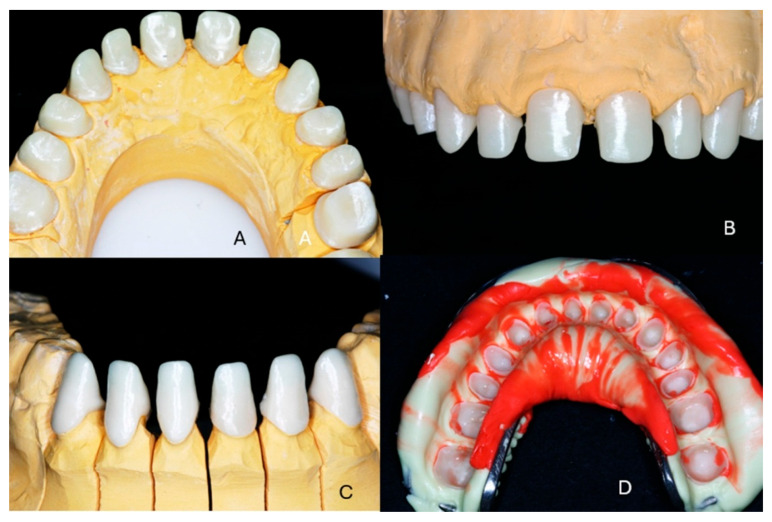
(**A**,**B**) The zirconia frameworks for the maxillary teeth. (**C**) The zirconia frameworks for mandibular anterior teeth. (**D**) Transfer impression.

The zirconia frameworks were tried by the patient, and a transfer impression was obtained for fabrication of a new working cast with details of the interdental papillae (Figure 7).

The zirconia frameworks were veneered in full shape with zirconia-compatible ceramic (Vita VM9, Vita Co, Bad Saeckingen, Germany) and tried by the patient. The fabrication procedure was accomplished first for the maxillary teeth using the provisional restorations of the mandibular arch as opposing teeth. The same procedure was followed for the mandibular teeth.

**Figure 8:**
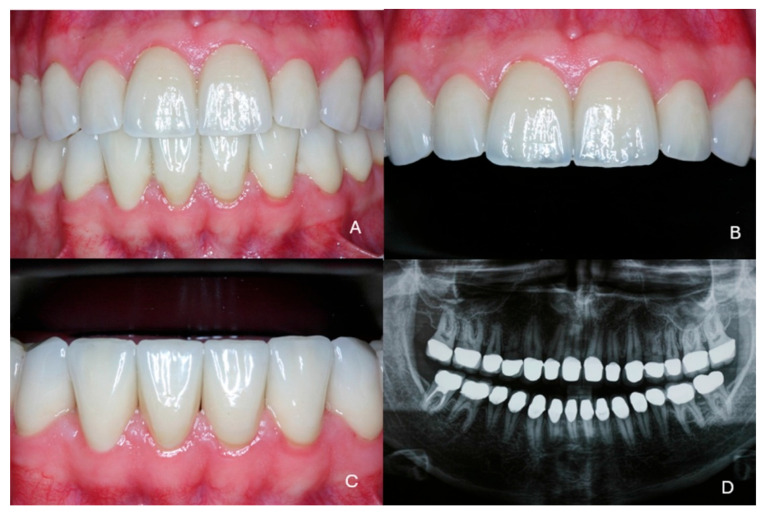
The final clinical situation at the end of the treatment. (**A**) Frontal view, (**B**) maxillary anterior teeth, (**C**) mandibular anterior teeth, (**D**) radiographic examination.

Before cementation the restorations were sandblasted on the internal side with 110 μm alumina particles at 2.5 bars for 15 s. The restorations were cemented using full-etch technique and dual-polymerization resin cement (Panavia F 2.0 resin cement, Kuraray Co., Tokyo, Japan) after application of ED Primer II on the teeth surfaces and Clearfil Ceramic Primer (both from Kuraray Co., Tolyo, Japan) on the interior surfaces of the crowns (Figure 8A–C). The radiographic examination at the end of the treatment confirmed the exact fit of the restorations (Figure 8D).

**Figure 9:**
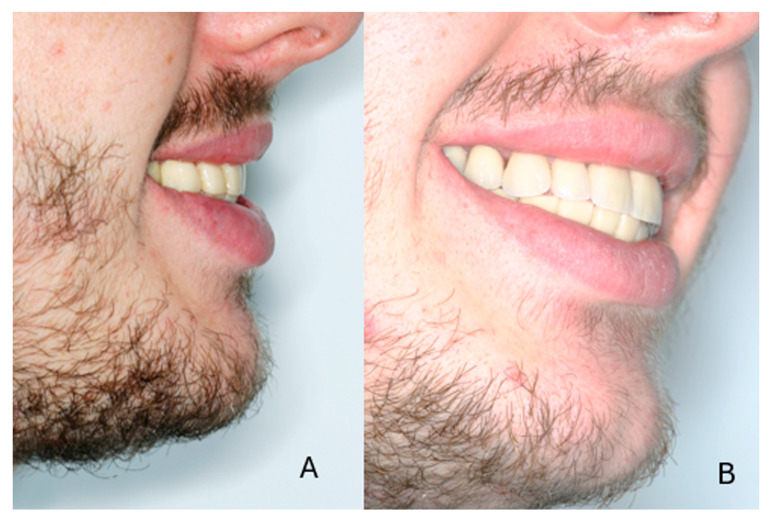
Extraoral photos of the patient at the end of the treatment. (**A**) Side view, (**B**) smile of the patient from the side.

***Final outcome/Patient perception:*** The patient was fully satisfied with the functional and esthetic outcomes of the treatment; his self-confidence and social life both significantly improved, as he mentioned in the first recall appointment a month after the end of the treatment (Figure 9).

**Figure 10:**
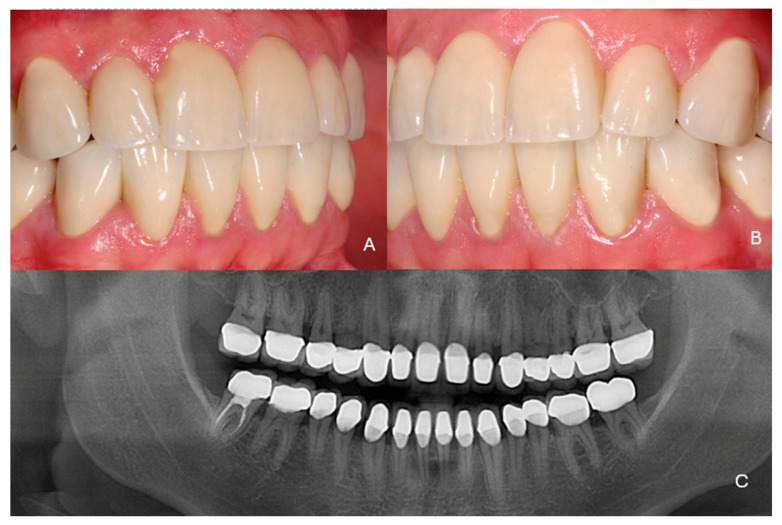
Clinical and radiographic examination at the 5-year recall. (**A**,**B**) Side views, (**C**) radiographic examination.

**Figure 11:**
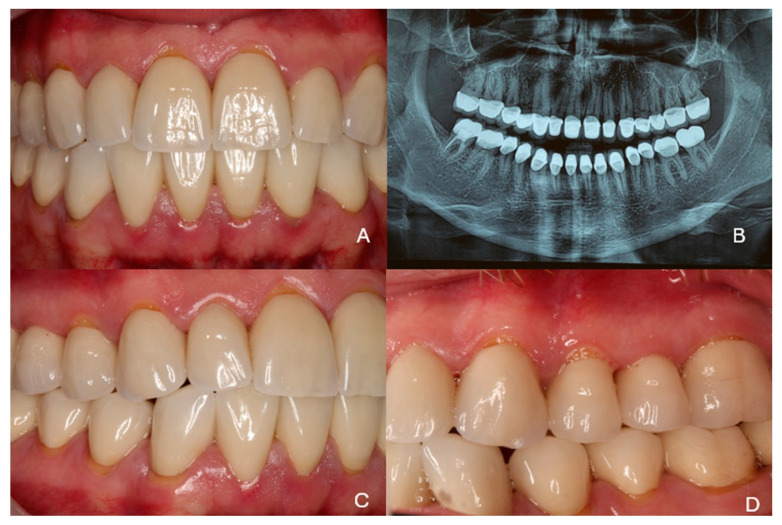
Clinical and radiographic examination at the 10-year recall. (**A**) Frontal view, (**B**) radiographic examination, (**C**,**D**) side views.

**Figure 12:**
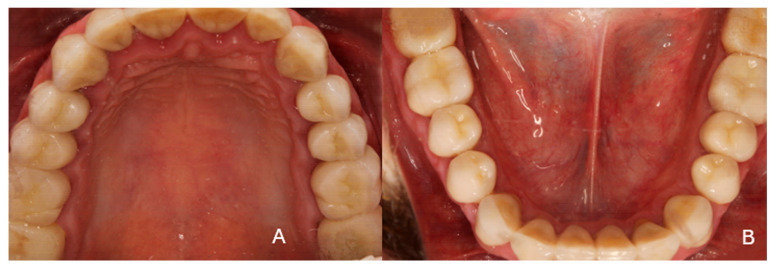
Occlusal photos at the 10-year recall. (**A**) Maxillary arch, (**B**) mandibular arch.

***Recalls:*** The clinical situation remained stable, as can be seen from the 5-year recall illustrated in Figure 10A–C. At the 10-year clinical and radiographic examination (Figure 11), a slight generalized gingival recession was observed because the patient did not follow a regular recall program due to a heavy schedule and several professional trips. Vigorous brushing and normal passive eruption of the teeth may have also contributed to the recession. The patient did not complain of sensitivity and did not wish any further treatment. The restorations did not show any signs of wear or discoloration after 10 years of clinical function (Figure 12).

## 3. Discussion

The treatment of patients with amelogenesis imperfecta (AI) is always a challenge for the clinician. The currently available evidence regarding treatment approaches for patients with AI is limited, and evidence from systematic reviews, randomized controlled trials, and from cohort studies, is not extensive. There is only one split-mouth study published on long-term outcomes of full-crown restorations in patients with AI using different crown materials [18].

Besides this clinical trial, few studies comparing different treatment procedures and assessing treatment outcomes in subjects with AI have been published, and the majority of available publications are case reports [17,19,20,21,22,23,24].

Various materials and techniques have been proposed for the prosthetic treatment of patients with amelogenesis imperfecta. Full-coverage crowns are usually necessary to cover the whole surface of affected teeth to restore the shape and surface. Metal–ceramic restorations have been used extensively, and have been the standard of care for years [4,22,25,26,27,28].

The use of all-ceramic restorations has been proposed by several authors in case reports, either with zirconia frameworks or with lithium disilicate [15,18,29,30,31,32].

In the present study, veneered crowns on zirconia frameworks were used because at the time of the treatment this would ensure the best combination of strength and esthetic outcome. Today, the use of new materials such as monolithic zirconia with increased translucency might also have been an option based on the favorable properties of such materials. At the time of the treatment, monolithic crowns showed a uniform color without any “depth illusion”. For this reason they were avoided. Currently, the new zirconia materials with increased translucency could be used, especially for the posterior teeth. However, there are no clinical trials supporting their use in similar cases.

Other materials like composite crowns and composite veneers have been proposed [33,34,35,36,37]. Their use, however, is mainly indicated as an interim restoration or limited to very young patients.

In general, the longevity of conservative dental restorations in patients with amelogenesis imperfecta (AI) is considerably reduced, and this reduction correlates with the severity of AI. In a comparison with a control group of patients with unaltered enamel (80%), the survival rate for coronal restoration in patients with AI was only 50% after 5 years, while the rate of replacement of defective restorations was about 2.5 times higher than in unaffected patients [38]. The main reason for the failure of restorations in patients with AI was fracture of the restoration or of the tooth, while in the control group without AI the main reason for restoration failure was recurrent caries. Compared with the control group (27%), the risk of either fracture of the restoration or fracture of hard tissue was substantially increased, to 60–69% [38].

Case reports of full mouth rehabilitations using all-ceramic materials with encouraging outcomes have been published [39,40]. The use of all-ceramic materials for patients with AI has only recently been supported by clinical trials or systematic reviews.

In a long-term retrospective clinical study [41] of all-ceramic crowns on patients either with or without amelogenesis imperfecta, with follow-up at 16.5 years, the all-ceramic crowns showed excellent results. The overall mean survival rate of single-tooth restorations was estimated to be 99.4% at 5 years and 91.4% at 10 years. The overall mean success rate was estimated to be 92.6% at 5 years and 81.4% at 10 years, these lower values being mainly due to chip-offs and crack formation. The mean annual failure rate (AFR) ranged between 1.5 and 2% over the years, but non-AI patients were affected more frequently by early technical complications in the facial veneering of anterior teeth, resulting in an AFR ranging between 5.2 and 4% [41]. The excellent longevity observed for full-coverage restorations could be possibly attributed to the extensive removal of the irregular AI-affected enamel layer in circumferential preparations.

In a systematic review based on six prospective and retrospective studies, indirect restorations showed better longevity and lower rates of complications compared to direct restorations. The use of ceramic materials for full-coverage crowns can be considered as a safe treatment option. Clinical studies on patients with all-ceramic restorations have shown very good results regarding longevity of the prostheses. In a prospective split-mouth trial, more than 97% of all-ceramic crowns inserted showed clinical quality which was at least satisfactory after 2 years of observation, with no significant differences observed between two types of all-ceramic restorative materials: veneered zirconia, and lithium disilicate ceramic [18]. Similar results were published in a later study [41], and ceramic restorations were also reported as the treatment choice for patients with amelogenesis imperfecta in a recent systematic review [42].

A detailed treatment plan customized for each clinical case is of crucial importance for a successful outcome. In the presented case, the first orthodontic treatment was accomplished without any prosthetic consultation and without predicting the final result. Consequently, the patient should undergo additional treatment with time and effort, while delaying the final outcome. A similar case report involving a similar approach has been published. In this case, a second opinion from a different orthodontist radically changed a treatment plan which had remained stable for 35 years [43].

Another case report with a long-term follow-up has been published. In this case, the maxillary teeth had been restored with partial-coverage ceramic restorations (veneers and overlays) while veneers and composite restorations had been used in the mandible [44]. In recent years, there have been some publications on the treatment of patients with amelogenesis imperfecta, but apart from case reports most publications have focused on associations with certain genes or combinations with other pathological conditions [45,46,47,48,49]. There is still no standardized protocol for treatment of such patients [50].

A limitation of the present case report is that it refers to a single patient and allows no generalized conclusions. However, it does show that under certain clinical conditions all-ceramic restorations can be considered as a viable treatment option.

Reviewing the case critically, ten years after the treatment and through the prism of currently available knowledge, some treatment options might be reconsidered. Extraction of the third molars, previously widely accepted as a treatment stage at the end of orthodontic treatment is nowadays doubted by several authors, with support from investigators and other research groups [51,52,53]. The use of partial-coverage restorations (veneers) or full-coverage crowns with thin cervical margins using lithium disilicate ceramics might also be considered as a treatment option to minimize dental tissue sacrifice [54,55,56,57].

**Clinical relevance:** Restorations in patients with amelogenesis imperfecta present a challenge to the clinician. For each patient, a detailed treatment plan should be proposes after a multidisciplinary evaluation of the patient. The age of the patient, the planned esthetic outcome, and the longevity of the restorations should all be taken into consideration.

## Data Availability

The data presented in this study are available on request from the corresponding author due to privacy or ethical restrictions.
